# Association between Stigma and Anxiety, Depression, and Psychological Distress among Japanese Women Undergoing Infertility Treatment

**DOI:** 10.3390/healthcare10071300

**Published:** 2022-07-13

**Authors:** Rie Yokota, Tsuyoshi Okuhara, Hiroko Okada, Eiko Goto, Keiko Sakakibara, Takahiro Kiuchi

**Affiliations:** 1 Department of Health Communication, Graduate School of Medicine, The University of Tokyo, Tokyo 113-8655, Japan; 2 Department of Health Communication, School of Public Health, The University of Tokyo, Tokyo 113-8655, Japan; okuhara-ctr@umin.ac.jp (T.O.); okadahiroko-tky@umin.ac.jp (H.O.); gotoue-tky@umin.ac.jp (E.G.); tak-kiuchi@umin.ac.jp (T.K.); 3 Department of Social Psychology, Faculty of Sociology, Toyo University, Tokyo 112-8606, Japan; sakakibara@toyo.jp

**Keywords:** infertility, stigma, women, anxiety, depression, psychological distress, mental health, health communication

## Abstract

Japan has the highest number of cases of infertility treatment in the world. Studies have indicated that women undergoing infertility treatment feel stigmatized and suffer from psychological symptoms such as anxiety and depression. However, in Japan, few studies have quantitatively examined the association between the stigma of infertility and psychological symptoms, and, to our knowledge, no study has examined its association using a scale with tested reliability and validity. This study aims to quantitatively examine the relationship between infertility stigma and anxiety, depression, and psychological distress among women undergoing infertility treatment, using a scale that has been validated for reliability and validity. The cross-sectional study was conducted in December 2021 through a web-based survey of 254 participants undergoing infertility treatment. A multiple regression analysis was performed to examine the relationship between stigma and anxiety, depression, and psychological distress. Stigma was a statistically significant predictor of anxiety, depression, and psychological distress (standardized β = 0.58, *p* < 0.001; β = 0.50, *p* < 0.001; β = 0.62, *p* < 0.001, respectively) after controlling for sociodemographic and infertility characteristics. Future studies should examine the causal relationship between stigma and anxiety, depression, and psychological distress and how to intervene to reduce stigma among women undergoing infertility treatment.

## 1. Introduction

Infertility is defined as the failure to conceive despite 12 months of unprotected intercourse [1]. Approximately 186 million people are infertile worldwide [2]. In Japan, 18.2% of couples (1 in 5.5 couples) have undergone or are currently undergoing infertility treatment or testing [3]. In 2017, in Japan, 56,617 infants were born through assisted reproductive medicine, that is, in vitro fertilization (IVF) and intracytoplasmic sperm injection (ICSI) [4]. This represents approximately 6.0% of all births in Japan in 2017 [5]. In 2017, approximately 450,000 infertility treatments were performed in Japan, making it the first country in the world in terms of utilization frequency [6].

In general, patients who are struggling to conceive express feelings of depression and anxiety [7]. The psychological symptoms of infertile women have been found to be equivalent to those of patients diagnosed with cancer [8,9]. In Japan, patients with infertility are highly anxious and depressed [6,10]. In a study published in 2021 in Japan, 55% of women participants undergoing advanced assisted reproductive technology, such as IVF or ICSI, showed more than mild depression symptoms, and 39% were categorized as highly anxious (state) [6]. Psychological stress caused by infertility can be attributed to a variety of factors, including uncertainty about the cause of infertility, uncertainty about the duration of treatment, and financial stress [10,11,12,13].

In addition to these factors, studies have shown that the stigma of infertility has negative impacts on health, including psychological distress such as anxiety and depression, low quality of life, and social isolation [14,15,16,17,18,19,20,21,22,23,24,25,26,27,28,29,30]. Stigma refers to the socially constructed process by which a group of individuals are labeled with socially undesirable attributes. These individuals are often devalued by the “whole and usual person” due to attributes and behaviors that are regarded as socially “deeply discrediting” [31]. Those who identify with or belong to a stigmatized group internalize the negative public perception of that group [32]. Furthermore, people with stigmatized identities tend to experience shame and discrimination because of their perceived inability to meet social expectations [33].

In Japan, a qualitative study indicated that the traditional beliefs that “married couples should have children” and the notion that “couples are fully fledged when they have a child” are deeply rooted in the country [34]. In fact, in a survey in 2018, 24.7% of married women answered that a couple is socially acceptable only after having a child [35]. According to a survey conducted in 2015, 75.4% and 67.4% of never-married men and women, respectively, responded that a couple should have a child after marriage [3]. Thus, in Japan, there is a socially accepted belief and expectation that one should have a child after marriage. Qualitative research has shown that some women undergoing infertility treatment internalize these values [36,37] and are obsessed with the idea that has been drilled into them that motherhood is a necessity, giving it topmost priority while considering their own existence as individuals as hardly important [38]. Although the stigma of women undergoing infertility treatment in Japan has been examined in qualitative studies, to the best of our knowledge, it has not been studied quantitatively.

In other countries, the relationship between stigma related to infertility and psychological distress, such as anxiety and depression, has been quantitatively examined [12,15,17,18,22,23,24,25]. According to Chinese, Turkish, and American research, stigma was significantly positively associated with psychological distress [15,19,22,23,25]. American and Indian research studies also examined the social pressure of “becoming a mother” as a predictor of distress [12,17]. In addition, psychological distress, such as anxiety or depression, was significantly positively associated with stigma and negatively associated with perceived social support [18,20]. However, to our knowledge, no study has examined the association between stigma and psychological distress using a validated measure in Japan. Only one study examined the association between psychological distress and feelings of inferiority due to not having children in a univariate analysis in Japan and indicated the existence of pressure to have a child [39]. However, this study examined only one aspect of stigma using originally created questions and did not address the relationship between stigma and psychological distress. Furthermore, this study was conducted approximately 20 years ago, and the trend may have changed in the present day. 

Previous studies in other countries also demonstrated the need for psychological intervention and suggested viable specifics for such interventions by examining the association between stigma and psychological distress, as well as quality of life [15,16,17,21,25]. In Japan, however, since these associations have been barely examined, the need for psychological interventions that would reduce stigma is not clear. Identifying the association between stigma and anxiety, depression, and psychological distress, could indicate the need for interventions in order to reduce stigma and motivate research on interventions in Japan. Similarly, in the context of the patient–healthcare professional relationship in clinical settings, this study may facilitate the understanding of the situation of women undergoing infertility treatment.

To overcome these limitations and fill the knowledge gap, we recently developed a reliable and validated scale to measure infertility stigma in Japan [40]. This study aims to quantitatively examine the relationship between infertility stigma and anxiety, depression, and psychological distress among women undergoing infertility treatment, using a scale that has been validated for reliability and validity.

## 2. Materials and Methods

### 2.1. Study Design and Setting

The purpose of this cross-sectional study was to test the relationship between infertility stigma and anxiety, depression, and psychological distress, controlling for sociodemographic and infertility characteristics of women undergoing infertility treatment in Japan. 

This cross-sectional study was conducted in December 2021 as part of our previous study that examined the reliability and validity of the Japanese version of the Infertility Stigma Scale [40], using a convenience sampling method. The survey was conducted online. The participants as well as the scale items in this cross-sectional study were identical to those in the scale development study [40]. 

### 2.2. Participants and Procedures

The participants were recruited from among those registered in the databases of a survey company in Japan. Women living in Japan aged 20–59 were selected from the database and were invited to participate in the survey. These women received an e-mail about the survey, and those who wished to respond could access the online survey company’s website. After logging in, potential participants in this study selected the survey for this study from the list of questionnaires and proceeded to the screening stage. A total of 100,208 individuals were sent the e-mail, of which 10,000 answered the screening questions online. Participants were selected for the screening survey on the basis of a set of inclusion and exclusion criteria. The inclusion criteria were those who answered the screening questions; were undergoing infertility treatment (excluding those undergoing infertility testing); did not have children; were native speakers of Japanese; were married (including common-law marriage); and gave consent to participate in this study. The exclusion criteria were individuals who answered affirmatively to screening questions asking if they had background or experience in healthcare, had a previous or current diagnosis of mental illness, or were experiencing secondary infertility. In total, 9734 participants were excluded from the screening questions.

A total of 266 respondents who agreed to participate were asked to complete a web-based survey on a computer or smartphone. The survey closed after 266 people completed it. Following this, data for this survey were received from an online survey company, which did not include the names, addresses, or e-mail addresses of the participants. The last question of the survey asked participants to “Please choose ‘uncertain’ from the following options” to determine whether they had properly read and answered the questions. From the 266 participants, we excluded nine who chose other than “uncertain” as lax respondents. Three participants who did not meet the eligibility criteria were also excluded; two did not undergo infertility treatment at the time of the survey, and one was pregnant. Finally, the data from 254 individuals were included in the analysis. The sample size for the multiple regression analysis should be approximately 20 times as large as the number of independent variables [41,42]. Thus, the sample size was determined to be approximately 220 women who met the study criteria, making 254 individuals an adequate sample size.

### 2.3. Ethical Considerations

This study was approved by the ethics committee of the University of Tokyo (approval number: 2021128NI). Informed consent was obtained from all the participants in accordance with the Declaration of Helsinki. 

### 2.4. Measures

In this study, the independent variables were infertility stigma (one variable), sociodemographic factors (six variables), and infertility characteristics (four variables). The dependent variables were anxiety, depression, and psychological distress (one variable for each). Infertility stigma in this study refers to an individual’s perception of stigma and self-stigma, such as feelings of shame and guilt. Anxiety and depression in this study refer to symptoms of anxiety and depression, and psychological distress is the aggregate of anxiety and depression symptoms. The following measures were administered in the survey.

#### 2.4.1. Sociodemographic Information and Infertility Characteristics

The participants were asked about their sociodemographic information, including age, duration of marriage, education, annual household income, occupation, and whether the couple lived with their parents or not. The participants were also asked about their infertility characteristics, including duration of infertility, duration of infertility treatment, causes of infertility, and treatment for infertility.

#### 2.4.2. The Japanese Version of the Infertility Stigma Scale (ISS)

The Infertility Stigma Scale was developed by Fu et al. in 2015 to assess individuals’ perception of stigma (perceived stigma) and feelings of loss of self-esteem, shame, and guilt (self-stigma) [43]. The scale consists of 27 items and includes items such as “I feel that I have an unfortunate fate”, “I am more sensitive to pregnancy and child because I can’t get pregnant”, and “It is common that people discriminate against infertile women”. Each item is measured on a 5-point Likert scale ranging from totally disagree (one point) to totally agree (five points), with higher scores indicating higher levels of stigma. The total score obtained from this scale ranges from 27 to 135. The Cronbach’s alpha coefficient for the original scale was 0.94. We recently developed the Japanese version based on the ISS developed by Fu and examined its reliability and validity [40]. The Japanese version of the scale, like the original, consists of 27 items, and the Cronbach’s alpha coefficient is 0.95 [40]. 

#### 2.4.3. The Japanese Version of the Hospital Anxiety and Depression Scale (HADS)

The Hospital Anxiety and Depression Scale (HADS) was developed by Zigmond and Snaith in 1983 to assess anxiety and depressive symptomatology [44]. HADS is widely used in infertility studies [10,39,45,46] and has also been used in many studies that examined the association between the stigma and psychological distress [18,47,48,49,50]. The scale consists of 14 items, with each item ranging from 0 to 3. The HADS includes items such as “Worrying thoughts go through my mind” and “I still enjoy the things I used to enjoy”. This scale can be divided into two subscales: anxiety and depression. Each subscale comprises seven items, with scores ranging from 0 to 21, with higher scores indicating greater anxiety and depression, respectively. The total score ranges from 0 to 42, with higher scores indicating greater psychological distress. A cut-off point of ≥8 was set to indicate doubtful cases of anxiety or depression, and a cut-off of ≥11 was set to indicate marked anxiety or depression. The Japanese version was translated by Kitamura et al. [51]. The reliability and validity of the Japanese version of the female workers were examined by Hatta et al., and the Cronbach’s alpha coefficients were 0.80 and 0.50 for anxiety and depression, respectively, in female workers [52]. In the present study, the Cronbachs’s alpha were 0.82, 0.81, and 0.68 for the total score, anxiety, and depression, respectively.

### 2.5. Statistical Analysis

Descriptive statistics were applied to determine the sociodemographic information and infertility characteristics of the participants. Categorical variables were summarized as percentages, and continuous variables were presented as means ± SD. We then examined the associations between sociodemographic information, infertility characteristics, stigma, anxiety, depression, and psychological distress by performing a two-sample *t*-test and a one-way ANOVA. After confirming that the distribution of residuals obtained in the model estimated using the Q-Q plot was close to a normal distribution, a multiple regression analysis was performed. We employed a multiple regression analysis using stigma, sociodemographic information, and infertility characteristics as independent variables and anxiety, depression, and psychological distress as dependent variables. Sociodemographic information and infertility characteristics were determined based on previous studies [15,16,20,21,53]. We employed the forced entry method, in which the factors to be input were predetermined. Data analysis was performed using R, version 4.1.1. Statistical significance was set at *p* < 0.05.

## 3. Results

### 3.1. Descriptive Statistics

The sociodemographic information and infertile characteristics of the participants are shown in Table 1. The participants had a mean age of 35.9 (SD = 5.5) years. The mean duration of marriage, infertility, and infertility treatment was 4.7 (SD 3.8), 3.3 (SD 2.9), and 2.3 (SD 2.4) years, respectively.

As shown in Table 1, the mean participant score on the ISS was 73.6 (SD = 20.9). Stigma was statistically significantly associated with the duration of infertility (*p* = 0.014) and the duration of infertility treatment (*p* = 0.002). However, stigma was not statistically significantly associated with education level (*p* = 0.059).

The mean values of participants’ scores on the HADS were as follows: 7.9 (SD = 4.3) for anxiety, 8.1 (SD = 3.7) for depression, and 16.0 (SD = 6.9) for psychological distress (HADS total). As for the prevalence and severity of anxiety and depression, 51.1% were anxious (anxiety score greater than 8 but less than 11:29.1%, anxiety ≥11:22.0%), and 54.0% were depressed (depression score greater than 8 but less than 11:28.0%, depression ≥11:26.0%). Anxiety was statistically significantly associated with living with parents (*p* = 0.014). Anxiety also had a statistically significant association with the level of education (*p* = 0.046). Anxiety was highest among participants with junior college or technical college graduate degrees and lowest among participants with graduate school degrees. Depression was not statistically significantly associated with sociodemographic information or infertility characteristics, while psychological distress was statistically significantly associated with living with parents (*p* = 0.007).

### 3.2. Multiple Regression Analysis

As shown in Table 2, stigma (standardized β = 0.58, *p* < 0.001) and living with parents (standardized β = 0.15, *p* = 0.004) statistically significantly predicted anxiety. The independent variables accounted for 36% of the variance of the dependent variable (adjusted R^2^ = 0.36).

As for depression, stigma (standardized β = 0.50, *p* < 0.001) and the duration of infertility (standardized β = −0.38, *p* = 0.004) statistically significantly predicted depression. The independent variables accounted for 24% of the variance of the dependent variable (adjusted R^2^ = 0.24).

Concerning psychological distress, stigma (standardized β = 0.62, *p* < 0.001) and living with parents (standardized β = 0.14, *p* = 0.006) statistically significantly predicted the psychological distress. The independent variables accounted for 39% of the variance in the independent variable (adjusted R^2^ = 0.39).

## 4. Discussion

We conducted a cross-sectional study to examine the relationship between stigma levels and the severity of anxiety, depression, and psychological distress among women undergoing infertility treatment. The results showed that stigma is a significant predictor of anxiety, depression, and psychological distress. It may be recommended that health professionals provide women undergoing infertility treatment psychoeducation to reduce self-stigma. Additionally, public initiatives should include an anti-stigma campaign for laypeople to reduce the stigma against infertile women face from the public. The main findings and implications are discussed as follows:

The mean score for the ISS of participants in this study was 73.6 (SD = 20.9), which is higher than that in the Turkish and Chinese studies [16,20,53,54]. This may be because secondary infertility was excluded from this study owing to Japan’s low total fertility rate of 1.36 in 2019 [55], making it difficult to consider secondary infertility as a socially deviant behavior. It is also known that the level of stigma is higher for primary infertility than for secondary infertility [14]. Therefore, the ISS score in this study may have been higher. Furthermore, the timing of this survey coincided with the period when public insurance coverage for infertility treatment was widely discussed in the media. This is because Japan’s declining birthrate has become an urgent issue as social security costs increase due to the aging of the population. The prevalence of reproductive medicine may cause women undergoing infertility treatment to believe that infertility should be cured [56]. Thus, it is possible that women undergoing infertility treatment may internalize the value of motherhood. This trend is likely to become even more noticeable as infertility treatment is now covered by public insurance, effective on 1 April 2022.

A univariate analysis showed a significant relationship between the degree of stigma and the duration of infertility and infertility treatment. These results are consistent with those of Chinese studies on stigma and quality of life among women undergoing IVF [16]. This may be due to the long period of infertility, which reduces hope for conception [16]. In contrast, this study found no association between higher education level and lower levels of stigma. This result was not consistent with the results of many previous studies in China, Turkey, and southern Ghana [14,16,20,53]. Although stigmatization depends on the availability of social, economic, and political power [57], Japan’s higher education enrollment rate in 2019 was 82.8% [58], indicating that education level may not be connected to power.

The mean values of the participants’ scores of the anxiety, depression, and psychological distress in this study were 7.9 (SD = 4.3), 8.1 (SD = 3.7), and 16.0 (SD = 6.9), respectively. Compared to previous Japanese studies using the HADS (anxiety: 5.7, SD = 2.7; depression: 4.6, SD = 2.9; psychological distress: 10.3, SD = 4.9), these mean values were all higher [10]. As for the prevalence and severity of anxiety and depression, 51.1% and 54.0% were categorized as more than doubtful cases of anxiety and depression, respectively. According to a study of patients with IVF or ICSI using the State–Trait Anxiety Inventory and the Quick Inventory of Depression Symptomatology—Self Report published in 2021, 39% and 55% of participants were categorized as having more than high anxiety (state) and more than mild depression, respectively [6]. Thus, a high percentage of participants in this study were categorized as having more than doubtful cases of anxiety. This may be partly related to COVID-19, which has caused increased anxiety [59]. During the COVID-19 pandemic, women tended to engage in precautionary behaviors, such as staying at home [60]. As a result, participants may have been socially isolated. 

Univariate and multiple regression analyses showed a significant relationship between living with parents and anxiety. Living with their parents may increase women’s anxiety by making them feel more pressured to have children. Moreover, the pressure may be accentuated with more people staying at home because of the COVID-19 pandemic. 

A univariate analysis showed no significant association between duration of infertility and depression, but a multiple regression analysis showed that duration of infertility was significantly associated with lower levels of depression. Participants in this study were not selected by strict random sampling from the population. Therefore, it is possible that a sampling bias may have occurred during the inclusion of participants and consequently affected the results of the analysis.

Multiple regression analysis in the present study found that stigma was a significant predictor of anxiety, depression, and psychological distress. These results are consistent with those of previous studies [15,18,19,20,22,23,25]. The standardized β for stigma was greater than that for the other variables. In other words, the predictive power of stigma was stronger than that of other variables that were statistically significant. One reason for this strong association can be explained by a qualitative study conducted in Japan, which showed that a large part of the connotations of the infertility experience involves the stigmatizing process, which can be distressing [36]. Some infertile women in Japan believe that marriage and motherhood are the normal paths for women. Childless people are regarded as deviants, the stigma of which they are unable to escape from [36,37].

The model of stigma-induced identity threat may explain why stigma is a predictor of anxiety and depression [61]. Women’s perception of being devalued by others in the dominant culture, social cues that they are in danger of being devalued, and perceived ease of stigma may lead to a sense that their identity is threatened, leading to anxiety and depression when coping is ineffective [61]. Women undergoing infertility treatment in Japan experience negative social interactions, including insensitivity [62]. Negative social interactions include others prying whether one has children or not, making uncalled-for remarks when the topic of conversation is children, acting insensitively and carelessly toward childless women, and showing disappointment or criticism about childlessness [62]. Thus, negative interactions in Japan may threaten the identity of women undergoing infertility treatment, resulting in anxiety and depression. Furthermore, the higher the level of stigma, the lower the perceived availability of social support, which may further isolate infertile women and increase their distress [18].

To reduce the self-stigma of women undergoing infertility treatment and the public stigma against infertile women, the following initiatives may be necessary. First, narrative enhancement and cognitive therapy interventions may be needed. These include experimental learning, positive change in experience of self, acquiring cognitive skills, enhanced hope, and coping and emotional change [63,64]. Similarly, interventions designed to raise awareness and defuse the legitimacy of discrimination may also be important in helping infertile women reduce stigma [65]. Future studies on psychological interventions are encouraged to reduce self-stigma in infertile women. Second, it may be necessary to conduct anti-stigma campaigns to reduce public stigma [66]. For example, framing strategies can help to redefine and de-stigmatize infertility [67]. A frame is a point of view that focuses on a particular part of the problem and ignores the remainder [67]. It may be necessary to present messages in the media and elsewhere that refute elements of the stigmatizing frame and undermine its narrative or messages that introduce a new frame and perspective without reference to the stigmatizing frame [66,67]. Future studies with anti-stigma campaigns are encouraged to reduce the public stigma against infertile women. As infertility treatment has been covered by insurance since April 2022, more women are expected to undergo infertility treatment in the future. Thus, in an environment where childbearing is a policy challenge, that is, childbearing is considered a “national context,” stigma-reducing programs will become increasingly important.

The present study has several limitations. First, this study was conducted during the COVID-19 pandemic. Therefore, it was not possible to recruit participants from hospitals and clinics. Because the survey was conducted via the Internet, participants diagnosed with mental illnesses could not be included because of difficulties in providing post-survey psychological care. Since many studies on infertility stigma also remove those diagnosed with mental illness as an exclusion criterion [12,15,16,21,22,25], it is not known how this exclusion criterion would have affected the results of our study. Hence, caution should be exercised in interpreting and generalizing the results. However, even after excluding those diagnosed with mental illness, an association between infertility stigma and anxiety, depression, and psychological distress was still observed. This study is important in that it demonstrates the need for efforts to reduce stigma in the primary prevention of stress-related symptoms among women undergoing infertility treatment. Second, because this survey was conducted using monitors from a web-based survey company, participant selection bias and sampling bias must be considered when interpreting the study results. Those enrolled as the survey monitors may have different backgrounds from the population of women undergoing infertility treatment in Japan. In addition, the participants in this study may not be representative of the general population of women undergoing infertility treatment in Japan, since they received the study recruitment e-mail and voluntarily participated in the survey. For these reasons, the results of this study may not be generalizable to all women undergoing infertility treatment in Japan. However, unlike recruiting participants in a hospital or clinic, the social desirability bias may have been reduced. Third, the COVID-19 pandemic may have had a significant impact on the participants’ mental health [60]. In particular, preventive behavior and fear of infection of COVID-19 may have caused higher levels of anxiety and depression. Thus, our estimates may overestimate the prevalence of anxiety and depression. Future studies should be conducted similarly when the impact of the COVID-19 pandemic has decreased. However, since efforts to reduce public prejudice against infertility have just begun through the Public Relations Office of the government of Japan in December 2021 [68], it is meaningful that this study shows the association between stigma and anxiety, depression, and psychological distress among women undergoing infertility treatment at this time. Fourth, due to the cross-sectional nature of the data, it was not possible to draw conclusions about the direction of the identified associations. However, the relationships examined were based on several previous studies [15,18,21,22,23,25]. In addition, the direction of the identified associations was identical to a model of stigma-induced identity threat [61]. Future research using longitudinal data is required to eliminate the possibility of reverse effects. Despite these limitations, to our knowledge, this study is the first to quantitatively examine the relationship between stigma and psychological distress among women undergoing infertility treatment in Japan, using a validated scale and its important implications as mentioned earlier.

## 5. Conclusions

The present study found that stigma is a strong predictor of anxiety, depression, and psychological distress. Infertile women may be regarded as deviants from the normal pathway of marriage and childbearing, with no escape from such stigmatization. Infertile women may also perceive stigma, appraise their identity as being threatened, fail to cope, and may be anxious and depressed. The results indicate that efforts to reduce infertility stigma such as psychoeducation and cognitive therapy intervention could be essential for reducing anxiety, depression, and psychological distress in women undergoing infertility treatment. Future research should examine the process of generating stress responses due to infertility stigma, and based on this, interventions to alleviate stress-related symptoms such as anxiety and depression are needed. 

## Figures and Tables

**Table 1:** Sociodemographic information, infertility characteristics of participants and their associations with stigma, anxiety, depression, and psychological distress. (n = 254).

Item	n	%	Stigma (ISS)			HADS-Anxiety			HADS-Depression			HADS-Total (Psychological Distress)		
			Mean	SD ^a^	*p* Value	Mean	SD ^a^	*p* Value	Mean	SD ^a^	*p* Value	Mean	SD ^a^	*p* Value
Age:					0.244 ^b^			0.284 ^b^			0.142 ^b^			0.505 ^b^
20–29	36	14.2	75.3	20.7		8.6	4.6		8.7	3.0		17.2	6.7	
30–39	147	57.9	71.8	20.8		8.0	4.1		7.7	3.7		15.7	7.0	
≥40	71	28.0	76.6	21.1		7.3	4.4		8.6	3.8		15.9	7.0	
Education:					0.059 ^b^			0.046 *^,b^			0.400 ^b^			0.064 ^b^
Less than high school	1	0.4	27.0	NA		1.0	NA		4.0	NA		5	NA	
High school graduate	43	16.9	74.8	20.3		8.7	4.4		8.9	4.1		17.6	7.5	
Vocational school graduate	33	13.0	72.0	23.0		6.7	4.2		7.4	3.5		14.1	6.7	
Junior college or technical college graduate	44	17.3	76.4	21.9		9.0	4.5		8.4	3.5		17.4	6.8	
University graduate	125	49.2	74.1	19.9		7.7	4.1		8.0	3.6		15.7	6.7	
Graduate school graduate	8	3.1	58.1	16.0		6.1	3.9		7.1	3.3		13.2	6.2	
Annual household income:					0.308 ^b^			0.189 ^b^			0.552 ^b^			0.257 ^b^
Less than JPY 2,000,000	12	4.7	81.2	19.1		9.7	5.5		8.6	3.9		18.2	7.9	
JPY 2,000,000 to JPY 4,000,000	38	15.0	78.3	18.6		9.1	4.3		8.7	3.8		17.8	7.0	
JPY 4,000,000 to JPY 6,000,000	72	28.3	74.8	19.7		7.7	4.3		8.2	3.6		15.9	6.7	
JPY 6,000,000 to JPY 8,000,000	59	23.2	71.4	22.7		7.8	4.2		7.8	3.5		15.6	7.1	
JPY 8,000,000 to JPY 10,000, 000	33	13.0	69.9	22.0		6.9	3.9		7.2	3.4		14.1	6.2	
More than JPY 10,000,000	40	15.7	71.1	21.4		7.6	4.2		8.5	4.2		16.1	7.2	
Employment status:					0.853 ^c^			0.457 ^c^			0.513 ^c^			0.901 ^c^
Unemployed	80	31.5	74	20.7		7.6	4.7		8.3	3.8		15.9	7.4	
Employed	174	68.5	73.5	21.0		8.0	4.1		8.0	3.6		16.0	6.7	
Duration of marriage (years):					0.067 ^c^			0.750 ^c^			0.756 ^c^			0.713 ^c^
<5	171	67.3	71.9	19.9		7.8	4.2		8.1	3.6		15.9	6.9	
≥5	83	32.7	77.2	22.4		8.0	4.5		8.2	3.9		16.2	7.1	
Living with parents:					0.239 ^c^			0.014 *^,c^			0.065 ^c^			0.007 **^,c^
No	239	94.1	73.3	21.1		7.7	4.2		8.0	3.7		15.7	6.9	
Yes	15	5.9	78.7	16.2		10.9	4.3		9.7	3.1		20.5	5.8	
Duration of infertility (years):					0.014 *^,c^			0.973 ^c^			0.379 ^c^			0.629 ^c^
<5	205	80.7	71.7	19.3		7.9	4.1		8.0	3.6		15.9	6.8	
≥5	49	19.3	81.5	25.2		7.9	4.9		8.6	3.9		16.5	7.6	
Duration of infertility treatment (years):					0.002 **^,c^			0.607 ^c^			0.352 ^c^			0.827 ^c^
<3	190	74.8	71.3	20.7		8.0	4.4		8.0	3.6		16.0	7.0	
≥3	64	25.2	80.5	20.0		7.7	3.9		8.5	3.9		16.2	6.9	
Determinism of etiology:					0.368 ^c^			0.726 ^c^			0.512 ^c^			0.573 ^c^
No	117	46.1	72.3	20.7		8.0	4.4		8.3	3.6		16.3	6.9	
Yes	137	53.9	74.7	21.0		7.8	4.2		8.0	3.8		15.8	7.0	
Treatment for infertility:					0.738 ^c^			0.083 ^c^			0.832 ^c^			0.238 ^c^
Other than IVF and ICSI	134	52.8	73.2	20.5		8.3	4.5		8.2	3.6		16.5	7.3	
IVF and ICSI	120	47.2	74.1	21.3		7.4	4.0		8.1	3.7		15.5	6.6	
Total	254		73.6	20.9		7.9	4.3		8.1	3.7		16.0	6.9	

* *p* < 0.05, ** *p* < 0.01 ^a^ Standard deviation; ^b^ one-way ANOVA.; ^c^ two-sample *t*-test.

**Table 2:** Multiple regression analysis to predict anxiety, depression, and psychological distress. (n = 254).

Variable	B	SE ^a^	95% CI ^b^	Std β ^c^	t	*p* Value
**HADS—Anxiety**						
(Intercept)	1.38	1.95	(−2.47, 5.23)		0.70	0.482
Age (years)	−0.03	0.05	(−0.13, 0.06)	−0.04	−0.70	0.486
Education ^d^	−0.10	0.19	(−0.48, 0.27)	−0.03	−0.55	0.583
Annual household income ^e^	−0.12	0.17	(−0.45, 0.22)	−0.04	−0.69	0.492
Employment status ^f^	0.25	0.48	(−0.71, 1.20)	0.03	0.51	0.611
Duration of marriage (years)	0.12	0.11	(−0.09, 0.32)	0.10	1.12	0.265
Living with parents ^g^	2.66	0.92	(0.85, 4.48)	0.15	2.89	0.004 *
Duration of infertility (years)	−0.03	0.18	(−0.38, 0.32)	−0.02	−0.15	0.878
Duration of infertility treatment (years)	−0.32	0.18	(−0.67, 0.03)	−0.18	−1.81	0.072
Determinism of etiology ^h^	−0.45	0.44	(−1.32, 0.42)	−0.05	−1.02	0.308
Treatment for infertility ^i^	−0.41	0.49	(−1.38, 0.56)	−0.05	−0.84	0.403
Stigma	0.12	0.01	(0.10, 0.14)	0.58	11.11	<0.001 **
R^2^	0.39					
Adjusted R^2^	0.36					
F	13.95					
*p*	<0.001					
**HADS—Depression**						
(Intercept)	2.32	1.83	(−1.29, 5.93)		1.27	0.206
Age (years)	0.03	0.04	(−0.06, 0.11)	0.04	0.59	0.554
Education ^d^	−0.17	0.18	(−0.52, 0.18)	−0.06	−0.96	0.339
Annual household income ^e^	0.02	0.16	(−0.29, 0.33)	0.01	0.14	0.891
Employment status ^f^	−0.44	0.45	(−1.33, 0.45)	−0.06	−0.97	0.333
Duration of marriage (years)	0.10	0.10	(−0.09, 0.30)	0.11	1.04	0.298
Living with parents ^g^	1.37	0.86	(−0.33, 3.07)	0.09	1.59	0.113
Duration of infertility (years)	−0.49	0.17	(−0.82, −0.16)	−0.38	−2.90	0.004 *
Duration of infertility treatment (years)	0.29	0.17	(−0.04, 0.62)	0.19	1.75	0.082
Determinism of etiology ^h^	−0.62	0.41	(−1.43, 0.20)	−0.08	−1.49	0.137
Treatment for infertility ^i^	−0.24	0.46	(−1.15, 0.67)	−0.03	−0.52	0.601
Stigma	0.09	0.01	(0.07, 0.11)	0.50	8.74	<0.001 **
R^2^	0.27					
Adjusted R^2^	0.24					
F	8.23					
*p*	<0.001					
**HADS-Total** **(psychological distress)**						
(Intercept)	3.70	3.09	(−2.40, 9.80)		1.20	0.233
Age (years)	−0.01	0.08	(−0.16, 0.14)	−0.01	−0.09	0.929
Education ^d^	−0.28	0.30	(−0.87, 0.32)	−0.05	−0.91	0.362
Annual household income ^e^	−0.10	0.27	(−0.62, 0.43)	−0.02	−0.35	0.724
Employment status ^f^	−0.19	0.77	(−1.70, 1.31)	−0.01	−0.25	0.801
Duration of marriage (years)	0.22	0.17	(−0.11, 0.55)	0.12	1.32	0.187
Living with parents ^g^	4.04	1.46	(1.16, 6.91)	0.14	2.77	0.006 *
Duration of infertility (years)	−0.51	0.28	(−1.07, 0.04)	−0.21	−1.81	0.071
Duration of infertility treatment (years)	−0.03	0.28	(−0.58. 0.52)	−0.01	−0.11	0.915
Determinism of etiology ^h^	−1.07	0.70	(−2.45, 0.31)	−0.08	−1.53	0.128
Treatment for infertility ^i^	−0.65	0.78	(−2.18, 0.88)	−0.05	−0.84	0.403
Stigma	0.21	0.02	(0.17, 0.24)	0.62	12.19	<0.001 **
R^2^	0.42					
Adjusted R^2^	0.39					
F	15.64					
*p*	<0.001					

* *p* < 0.01, ** *p* < 0.001 ^a^ Standard error; ^b^ confidence interval (lower-bound, upper-bound); ^c^ standardized β; ^d^ 0 = less than high school, 1 = high school graduate, 2 = vocational school graduate, 3 = junior college or technical college graduate, 4 = university graduate, 5 = graduate school graduate; ^e^ 0 = less than JPY 2,000,000, 1 = JPY 2,000,000 to JPY 4,000,000, 2 = JPY 4,000,000 to JPY 6,000,000, 3 = JPY 6,000,000 to JPY 8,000,000, 4 = JPY 8,000,000 to JPY 10,000, 000. 5 = more than JPY 10,000,000; ^f^ 0 = unemployed, 1 = employed; ^g^ 0 = no, 1 = yes. ^h^ 0 = no, 1 = yes; ^i^ 0 = other than IVF and ICSI, 1 = IVF and ICSI.

## Data Availability

The data supporting the findings of this study are available from the corresponding author, R.Y., upon reasonable request. The data were not publicly available because of ethical considerations.
